# The First Protocol for Assessing Welfare of Camels

**DOI:** 10.3389/fvets.2020.631876

**Published:** 2021-01-28

**Authors:** Barbara Padalino, Laura Menchetti

**Affiliations:** Division of Animal Sciences, Department of Agricultural and Food Sciences, University of Bologna, Bologna, Italy

**Keywords:** camel, welfare, behavior, feeding, housing, health

## Abstract

The aim of this study was to develop and describe a protocol for assessing welfare in camels reared in intensive or semi-intensive systems. A literature review was conducted searching for scientific papers on assessment of animal welfare and camel behavior, management, physiology, and pathology. The paradigms of Five Freedoms, the Five Domains Model, and the welfare principles and criteria applied by the Welfare Quality® and AWIN methods were then adapted to camels. A combination of animal-, resource- and management-based indicators were selected and categorized according to three levels of assessment: (i) Caretaker, (ii) Herd, and (iii) Animal. The Caretaker level is an interview of 23 questions exploring the caretaker's background, experience, and routine management practices. The Herd level is a check of the herd and of the place (i.e., box/pen) where camels are kept. The Animal level is a visual inspection aiming at evaluating individual camel behavior and health status. The selected indicators are presented for each welfare principle and level; for instance for the principle of “Appropriate nutrition,” feeding management is investigated at Caretaker level; feed availability and quality, the number of feeding points, and camel feeding behavior are recorded at Herd level, while body condition score (BCS) is evaluated at Animal level. In this study recording sheets for the assessment at the three levels are proposed and how to conduct the assessment is described. Limitations of the proposed protocol are also discussed. Further applications of this protocol for assessing camel welfare on a large number of farms is needed to validate the proposed indicators and identify the thresholds for their acceptability as well as to develop overall welfare indices and welfare standards in camels.

## Introduction

Official FAO statistics report that there are over 35 million camels in the world (last update: 2018). Their number has grown by about 15% in a 10-year period and it is destined to progressively increase in the future (1). Although it is difficult to estimate the economic importance of this species, both present and future (2), some explanations of their growing popularity may be deduced. First of all, there are probably no other animal species as versatile for the human being: the camel is a multipurpose animal, used to produce meat, milk, wool, hides, and skins, with an active role in agricultural, cultural and recreational life of many populations worldwide (2, 3). In recent decades, several studies have confirmed the nutritional quality of camel products, particularly its milk (4–7), suggesting attractive marketing prospects and therapeutic uses (8, 9). Some modernizations in farming techniques, such as machine milking, have been introduced but room for improvement still exists (3, 10, 11). Genetic improvement and rational farm management could enhance the productive efficiency of camels and, therefore, their economic profitability. Finally, climate change and increasing desertification are likely to make camels' adaptive abilities more and more appreciated as they demonstrate peerless productive potential in arid conditions (3, 4).

Despite these promising prospects for camel rearing, there is still very little attention and knowledge about its welfare; these shortcomings concern both the scientific and legislative aspects. Recent bibliometric research (12) pointed out that, although the scientific interest in regards to the camel species has grown, little attention has been paid to camel welfare issues. There are still serious gaps of knowledge in camel physiology and behavior, in the impact of different housing systems on its welfare and relationship with humans. Specific indicators for assessing camel welfare have not been developed yet (12) and camels have been blatantly neglected by international legislation. The World Organization for Animal Health included camels in the document of recommendations for transport by land, but no specific chapters of “Terrestrial Code” addressed the welfare aspects of camels production systems (13). The first European project, named Welfare Quality® project, has focused on other species (14, 15) and the camel did not even appear in the second largest European project, the Animal Welfare Indicators Project (AWIN), which had to cover species not considered in the Welfare Quality® (16). The key idea of both Welfare Quality® and AWIN projects is that animal welfare is a multidimensional concept and multiple aspects of physical and mental health should be stated and evaluated accordingly. The latter protocols organize the welfare dimensions in principles and criteria extending the notions of the Five Freedoms (17) and suggest valid, measurable and reliable indicators for each criterion. The indicators have been further classified according to two generic approaches: (a) animal-based indicators (e.g., behavioral measurements, body conditions, health records); and (b) resource- and management-based indicators (e.g., space allowance, feeding regime, environmental characteristics) (18, 19).

Welfare Quality® and AWIN projects were aimed at developing assessment protocols that provided tools feasible and practical to evaluate animal welfare. Not only animals but also stakeholders would benefit from such a welfare assessment tool. A standardized tool could be used to evaluate individual resources (i.e., diet, housing), compare different husbandry systems, quantify a range for optimal welfare and assess farmers' compliance, develop quality certifications, identify welfare risk factors and give evidence for developing new animal welfare legislation (20).

Hypothesizing that camel welfare could be assessed using animal-based, resource- and management-based indicators and to fill the aforementioned gaps of knowledge, this study was aimed at introducing an innovative protocol for assessing welfare in camels reared in intensive or semintensive farming system conceived by the idea of adapting criteria and principles of Welfare Quality® and AWIN protocols to this peculiar species.

## Materials and Methods

### Selection of Indicators

A group of researchers with experience in camel behavior and animal welfare reviewed the relevant scientific literature to select promising indicators to be included in the protocol. Research databases (PubMed, Web of Science, Google Scholar, and Scopus) were selected and the search was refined limiting the search to recent academic journal articles describing assessment of animal welfare. Since the literature available on camels was very scarce, the researchers mainly referred to the indicators used for horses and ruminants according to the AWIN and Welfare Quality® protocols (15, 18, 21–23), evaluating which ones could be adapted to camels. A combination of animal-, resource- and management-based measures were preferred for inclusion in this protocol as commonly done in the literature (20). The list of indicators was further refined using the experience in the field of the researchers to cover all aspects of camel welfare and consulting articles published on camel physiology, ethology, husbandry, and pathology (24–41). The literature review included only papers written in English.

The selection of the welfare indicators also took into account the principles of validity, reliability, and feasibility as reported in the literature for other species (42, 43). Thus, the indicators that require further laboratory analysis (e.g., metabolic profiling) were excluded to meet the principle of feasibility. All invasive measurements or measurements involving physical contact with animals were also excluded as camels could be untamed making procedures stressful for animals and unsafe for operators. Moreover, although their potential importance in assessing animal welfare is recognized, data such as milk quality and quantity, fertility indexes, mortality or daily body gains were not included in this protocol because they are difficult to be directly verified by an assessor. Finally, only measurable indicators were chosen to comply with the principles of validity and reliability (42). After the aforementioned process, the indicators were organized accordingly with the four principles and 12 criteria developed by Welfare Quality® (14, 15, 18).

### Caretaker, Herd, and Animal Levels

**Figure 1:**
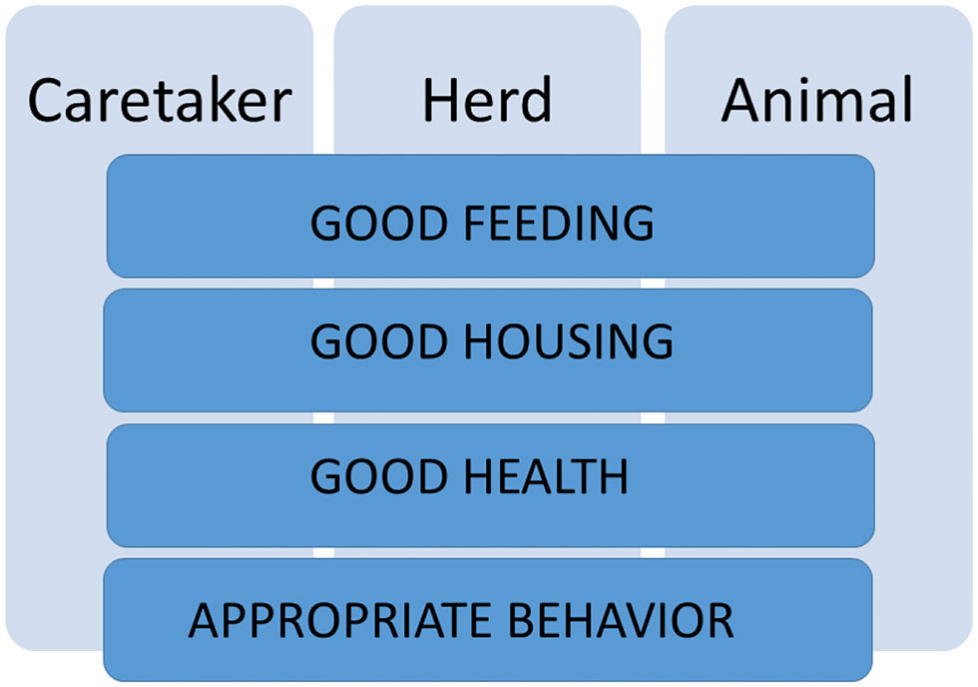
The welfare principles of Good feeding, Good housing, Good health and Appropriate behavior are evaluated at three levels: Caretaker level, Herd level, and Animal level.

Data related to all indicators included in each welfare principle should be collected at three levels depending on their origin: from the caretaker, “Caretaker level,” from the direct evaluation of a group of animals and the pen where they are kept, “Herd level,” or from the individual camel, “Animal level” (Figure 1). A recording sheet was developed for each level of assessment.

The Caretaker level is a face to face interview and comply with the principles of the Terrestrial Code according to which caretakers are responsible for the humane handling and care of the animals, and they should have sufficient skills and knowledge to ensure that animals are treated following animal welfare principles (13). The questions were selected and adapted from a questionnaire previously developed for investigating the knowledge of animal welfare among camel caretakers (44). Mainly resource- and management-based indicators were chosen for the Caretaker level embracing all principles (Tables 1, 2).

**Table 1:** Camel welfare indicators were selected by researchers for the principles of Good feeding and Good housing.

**Principles**	**Welfare criteria**	**Welfare indicators**		
		**Caretaker level**	**Herd level**	**Animal level**
Good feeding	Appropriate nutrition	Feeding management	Feed availability Feed quality	BCS
			Feeding points	
			Feeding behavior	
	Absence of prolonged thirst	Watering management	Water availability Water quality	Bucket test
			Water points	
			Drinking behavior	
Good housing	Comfort around resting	Years of experience in working with animals	Bedding Space allowance Rubbish	Resting behaviorInsects
			Resting behavior	
	Thermal comfort	Years of experience in working with camels	Temperature Humidity Wind speed Shade Use of the shade	Use of the shade
	Ease of movement	Camel exercise	Pen/box dimension	Tethering
			Tethering	Hobbled
			Fence quality	

*The indicators were divided according to welfare criteria and source of information (Caretaker level: indicators collected through an interview of the caretakers; Herd level: indicators collected by a direct evaluation of a group of animals and their pen/box; Animal level: indicators collected by a direct evaluation of individual camels)*.

*BCS, Body Condition Score*.

**Table 2:** Camel welfare indicators were selected by researchers for the principles of good health and appropriate behavior.

**Principles**	**Welfare criteria**	**Welfare indicators**		
		**Caretaker level**	**Herd level**	**Animal level**
Good health	Absence of injuries	Camel injury observed	Animals injured Type of injury	Injury Scar Swollen Joint Lameness
	Absence of disease	Camel disease observed Camel health check Medical treatments	Sick animals Type of disease	Disease Hair coat conditions Ectoparasites Discharge Diarrhea Abnormal udder Abnormal breathing Coughing
	Absence of pain and pain induced by management procedures	Caretaker's ability to identify pain	Animals in pain Animals with a nose-ring, cauterizations and wounds from halters or similar	Evident pain
Appropriate behavior	Expression of social behavior		Social behavior Aggressive behavior	Social interaction
	Expression of other behavior	Camel behavioral problems observed	Stereotypies Other abnormal behaviors	Stereotypies Other abnormal behaviors
	Good human-animal relationship	Experience in camel handling		Approaching test
		Caretaker's skills in identifying distress		
		Caretaker's knowledge of animal welfare		
	Positive emotional state			Behavior repertoire

*The indicators were divided according to welfare criteria and source of information (Caretaker level: indicators collected through an interview of the caretakers; Herd level: indicators collected by a direct evaluation of a group of animals and their pen/box; Animal level: indicators collected by a direct evaluation of individual camels)*.

The Herd level is a check of the herd and of the place (i.e., box/pen) where camels are kept. It includes robust and feasible indicators requiring no or minimal handling. Resource- and management-based indicators were chosen for the “Good feeding” and “Good housing” criteria (Table 1), while mainly animal-based indicators were chosen for the “Good health” and “Appropriate behavior” criteria (Table 2).

The Animal level consists of behavioral observation, behavioral tests, and a visual inspection of individual camels. Mainly animal-based indicators were chosen for all criteria. Among the measures proposed by the AWIN and Welfare Quality® protocols, only the most promising ones in terms of feasibility in the camel field were selected (e.g., BCS) (Tables 1, 2).

## Results

Each farm welfare assessment should start with a meeting with the camel farm manager/caretaker, for explaining the protocol. The farm welfare assessment should be carried out at a fixed time, for example, 10:00 a.m., respecting the farm's routine practices. The on-farm welfare assessment would be carried out with some steps taken from outside and other inside the box/pen where the animals are kept (Supplementary Figure 1).

### Camel Welfare Assessment at Caretaker Level

Table 3 shows the questions of the interview composed of 14 closed-ended and nine open-ended questions. During the interview, general information on the animals and their management is collected. In particular, the questions investigate the following aspects: demographic characteristics of the caretaker and camels, feeding and health management, self-evaluation of their ability to assess pain and distress, and knowledge of animal welfare. This information is aimed at double-checking the reported management with the data collected by the assessor at Herd or Animal level, verifying the caretaker's knowledge of welfare and at identifying possible hazards. For example, the caretakers' statements relating to the frequency of water distribution would be compared with the Herd level indicators of water quantity and quality. The criteria suggested by the caretaker to evaluate a camel in pain would indicate the ability to early quickly identify a camel that was suffering. Finally, the experience in camel handling and in managing other farm animals would affect farm management, health, and the human-animal relationship.

**Table 3:** Camel welfare recording sheet at Caretaker level.

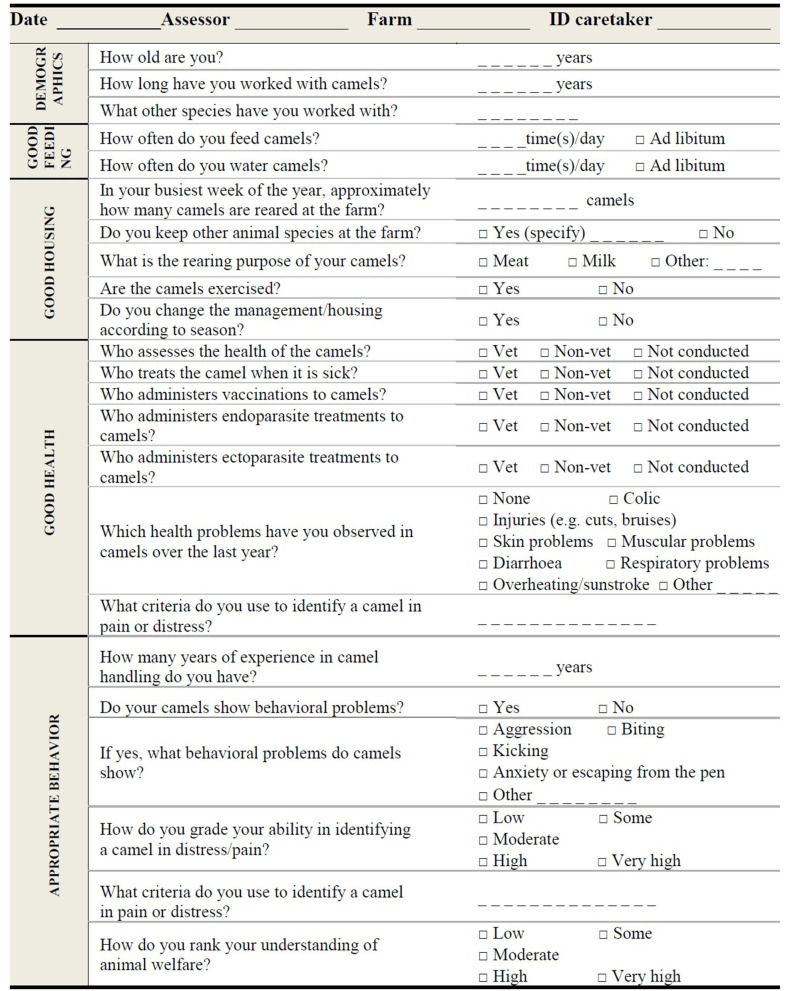

*Questions to pose during a face to face interview with the farm manager/caretaker divided according to the welfare criteria*.

### Camel Welfare Assessment at Herd Level

Tables 4–6 show the recording sheets for the assessment at Herd level. They are lists of parameters related to the environment, camel herd and the place where the camels are kept (i.e., pen); their collection should be carried out without disturbing the animals. The first measurements collected from outside the pen are related to animal behavior. After the census of the number of animals, the assessor should observe them and record the behaviors included in the Appropriate behavior section shown by each member of the herd during 3 min (45) (see scan sampling ethogram in Supplementary Table 1). Then, the environmental parameters, such as THI, and general characteristics of the pen/box, such as dimension and shape should be recorded. Instruments for detecting environmental parameters should be placed near the fence at the level of the camel's nose. Entrance into the pen is generally required to evaluate indicators of Good health, especially if there are many animals or very large pens, while it is always required to carry out the rest of the measurements and scoring included in the Good housing and Good feeding principles (e.g., dimension of feeding and drinking troughs). In addition to the facilities' dimension, their cleanliness should also be evaluated. The cleanliness of feeding and water points should be scored using a three-point scale: “dirty” if there is an abundant presence of organic or inorganic materials, such feces or debris, “partly dirty,” if the facilities are contaminated by a few foreign materials, or “clean” (Table 4). Furthermore, the position of the feeding and watering point (i.e., in the sun or in the shade) and the temperature of the drinking water should be noted. Bedding should be similarly evaluated, recording the type of bedding and its cleanliness according to the presence of feces or unsuitable material (Table 5). The Herd level assessment also requires a qualitative description of the fences and the rubbish present in the pen. In particular, the condition of the fences should be reported as a binary variable (broken/unbroken) while the rubbish should be scored as “No rubbish,” “Small,” “Medium,” and “Large” size according to its dimension (Table 5 and Supplementary Figure 2). Other indicators such as density and trough space, should be calculated at a later stage. A selection of camel boxes/pens to be assessed may be applied following the rules suggested by the AWIN protocol for goats (section 3.6.1) (46) and stratifying according to the category of animals kept in the pens (young, adults, pregnant, and stage of lactation). The selection of the pen should be randomly conducted excluding the pens used as infirmary, culling, and quarantine. Namely, if <2 pens were present at the farm, all pens would be assessed; if the farm had 3–7 pens, two pens would be assessed; if the farm had 8–10 pens, three pens would be assessed; finally, if the farm had more than 10 pens, 25% of the pens would be assessed.

**Table 4:** Camel welfare recording sheet for indicators of Good feeding collected at Herd level.

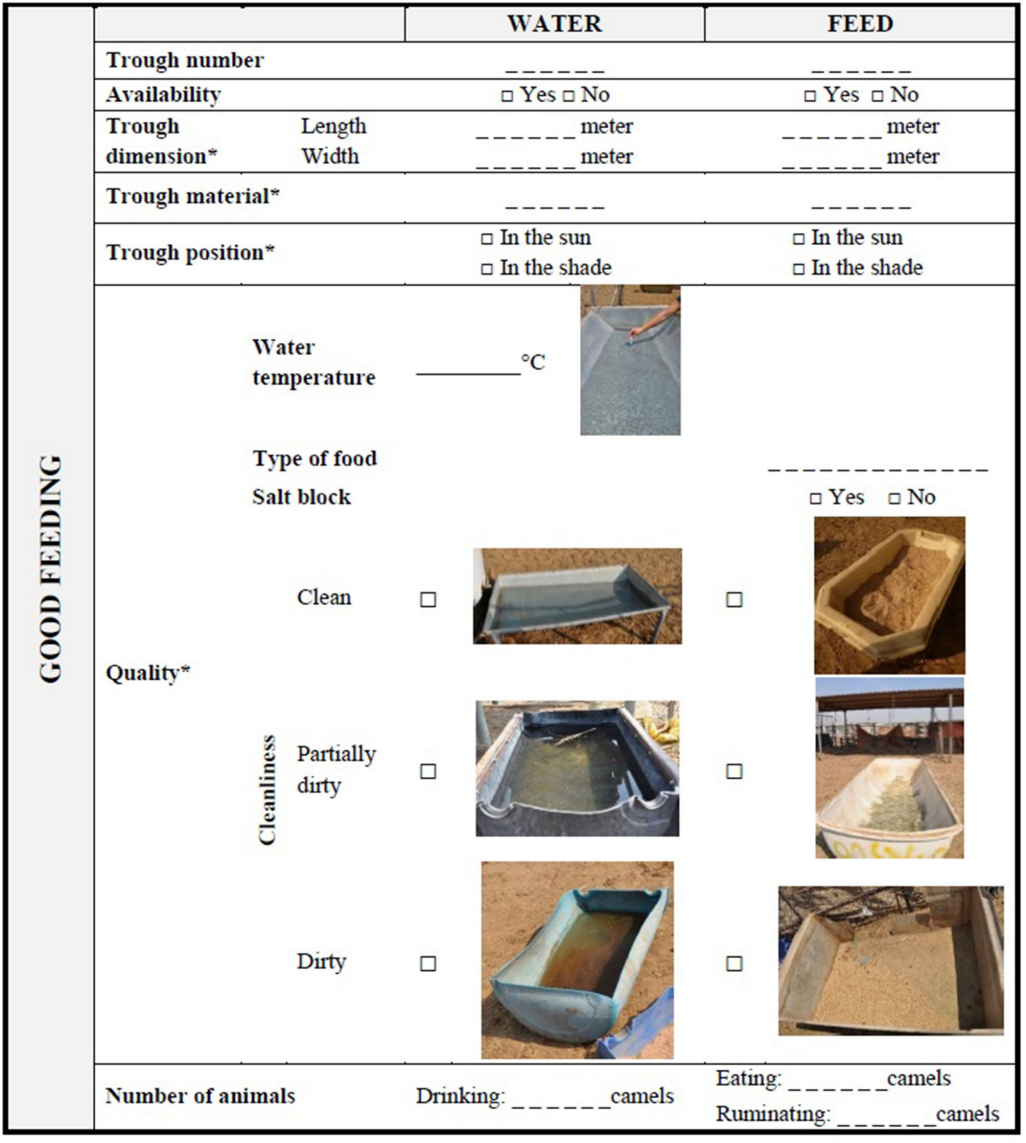

*The Herd level consists of a check of the herd and of the place where the camels are kept*.

*For each water/feeding point.

**Table 5:** Camel welfare recording sheet for indicators of Good housing collected at Herd level.

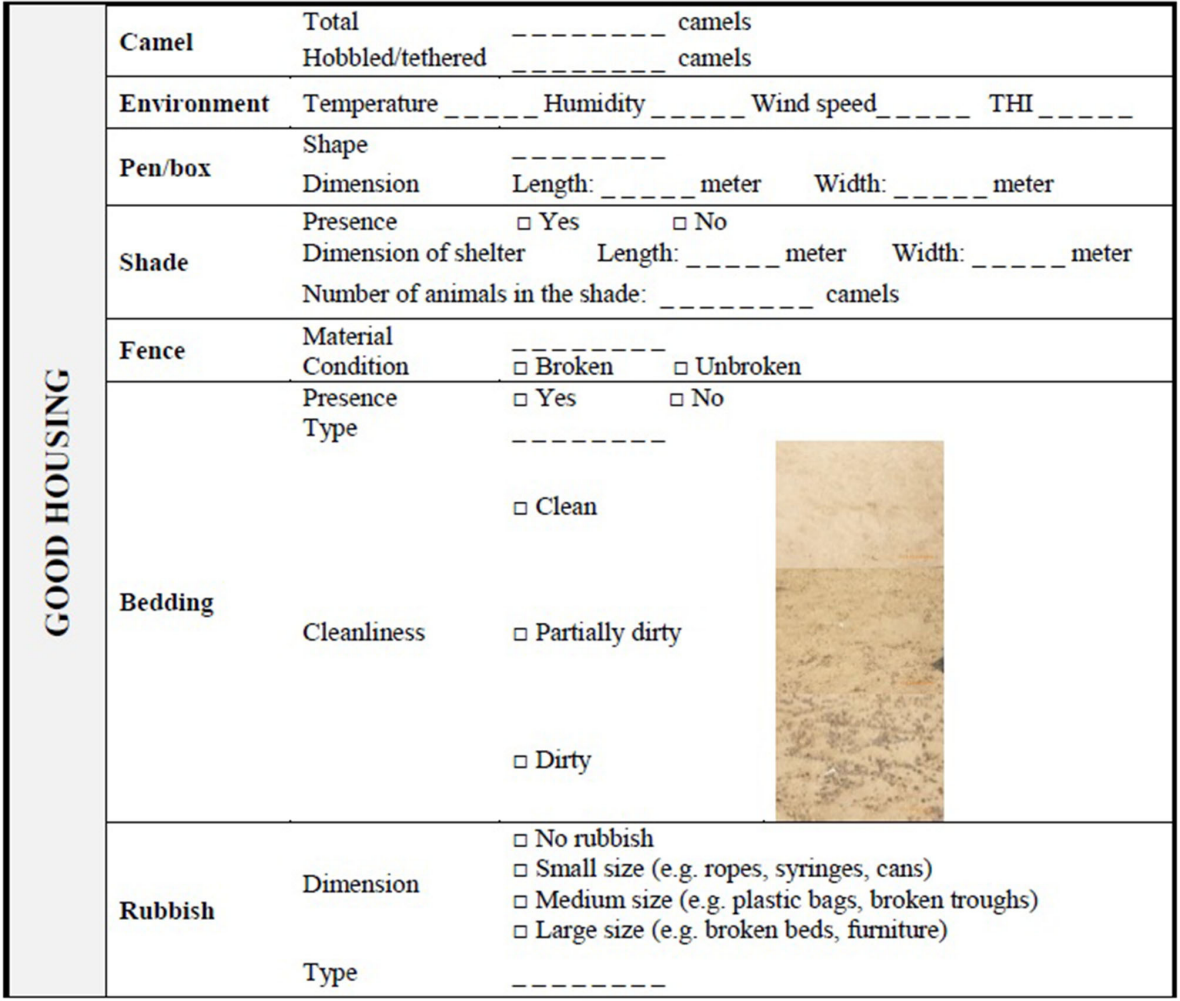

*The Herd level consists of a check of the herd and of the place where the camels are reared*.

**Table 6:** Camel welfare recording sheet for indicators of Good health and Appropriate behavior collected at Herd level.

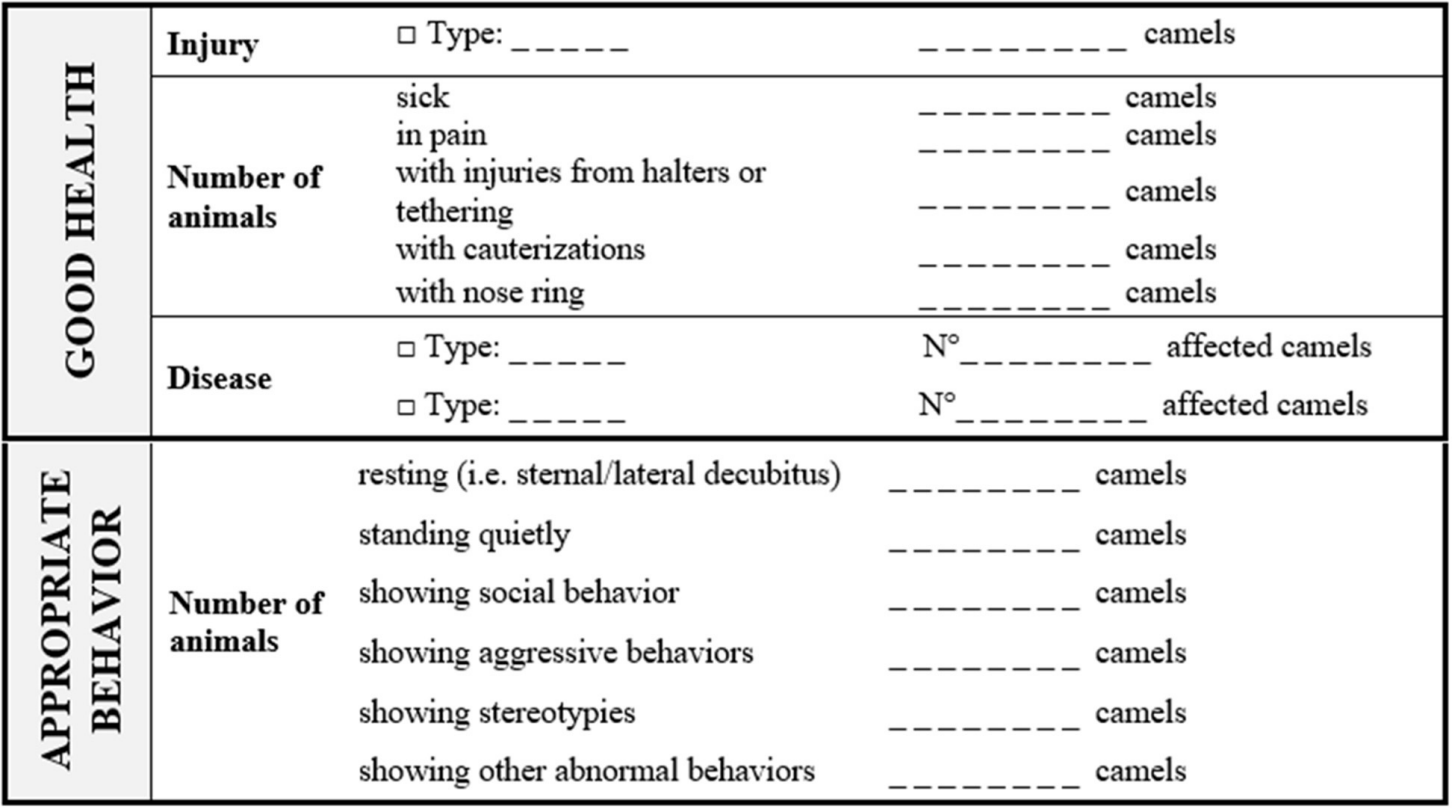

*The Herd level consists of a check of the herd and of the place where the camels are reared*.

### Camel Welfare Assessment at Animal Level

Tables 7, 8 show the recording sheets for the assessments at Animal level. The Animal level assessment involves a closer look and contact with the camel without any invasive procedures. The number of animals to be assessed should be chosen following the rules proposed by AWIN for goats' selection assuming a 50% prevalence, a confidence interval of 95%, and an accuracy of 10% (section 3.6.3) (46). However, to minimize the impact on camels, non-restrictive criteria, such as a level of confidence of 90% or less, or rules of thumb could be adopted.

**Table 7:** Camel welfare recording sheet for indicators of Good feeding collected at the Animal level.

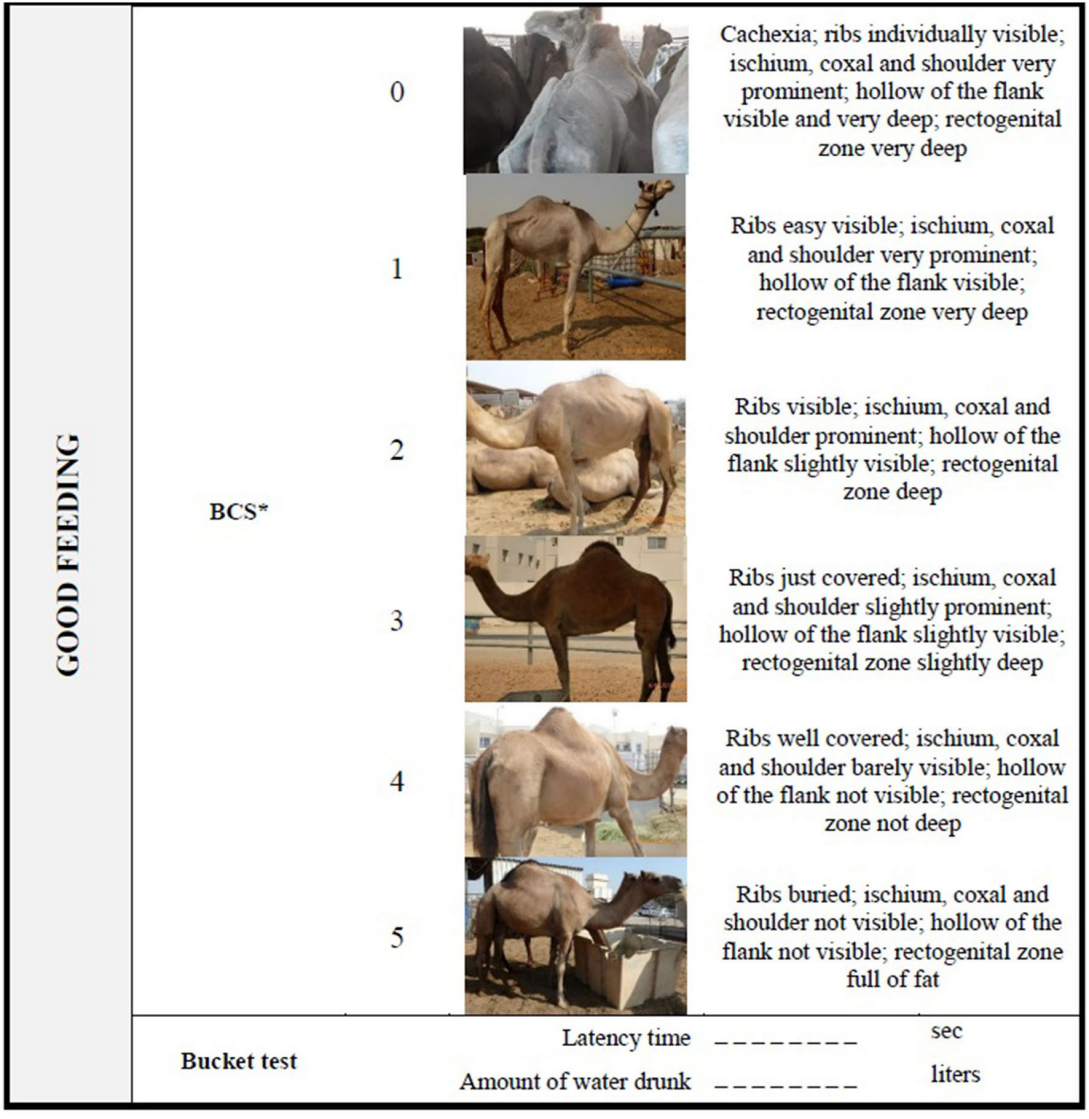

*The Animal level consists of a check of the individual animal and behavioral tests*.

* *BCS, Body Condition Score, adapted by Faye et al. (47)*.

**Table 8:** Camel welfare recording sheet for indicators of Good housing, Good health, and Appropriate behavior collected at the Animal level.

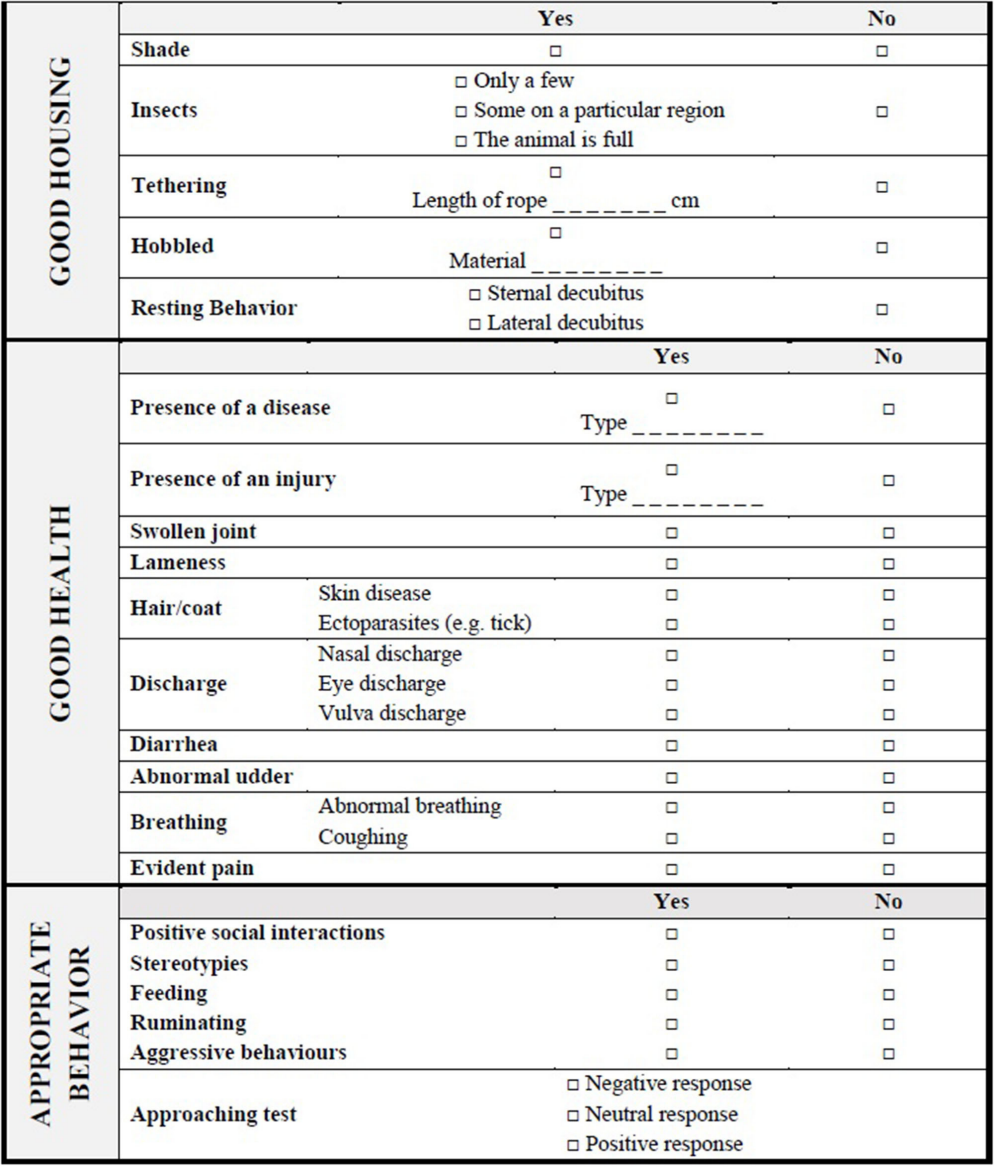

*The Animal level consists of a check of the individual animal and behavioral tests*.

Initially, a behavioral observation of 3 min (direct observation or video taking for further analysis) should be conducted from outside the pen without disturbing the animal. During the behavioral observation, the assessor should record parameters included in Good housing (e.g., position of the camel in the shade or the sun, the presence of insects, and physical restraint) and the other behavior traits included in the recording sheet (see ethogram in Supplementary Table 1) using the one-zero (occurrence or non-occurrence) sampling method (45). Then, an approaching test modified by Wulf et al. (48) should be performed (Supplementary Figure 3). Briefly, an unfamiliar test person (i.e., tester) enters into the pen where the camel is kept and approaches the camel slowly, one step at a time. The test is stopped if the camel shows avoidance or aggressive behavior (turning the head, running away, biting) or when the tester can approach the camel and put a hand close to the nose of the camel. The tester should be a person with a solid scientific background on animal behavior. The camel behavioral responses should be classified as “Positive,” “Neutral,” or “Negative” (Table 9).

**Table 9:** Camel approaching test scoring system.

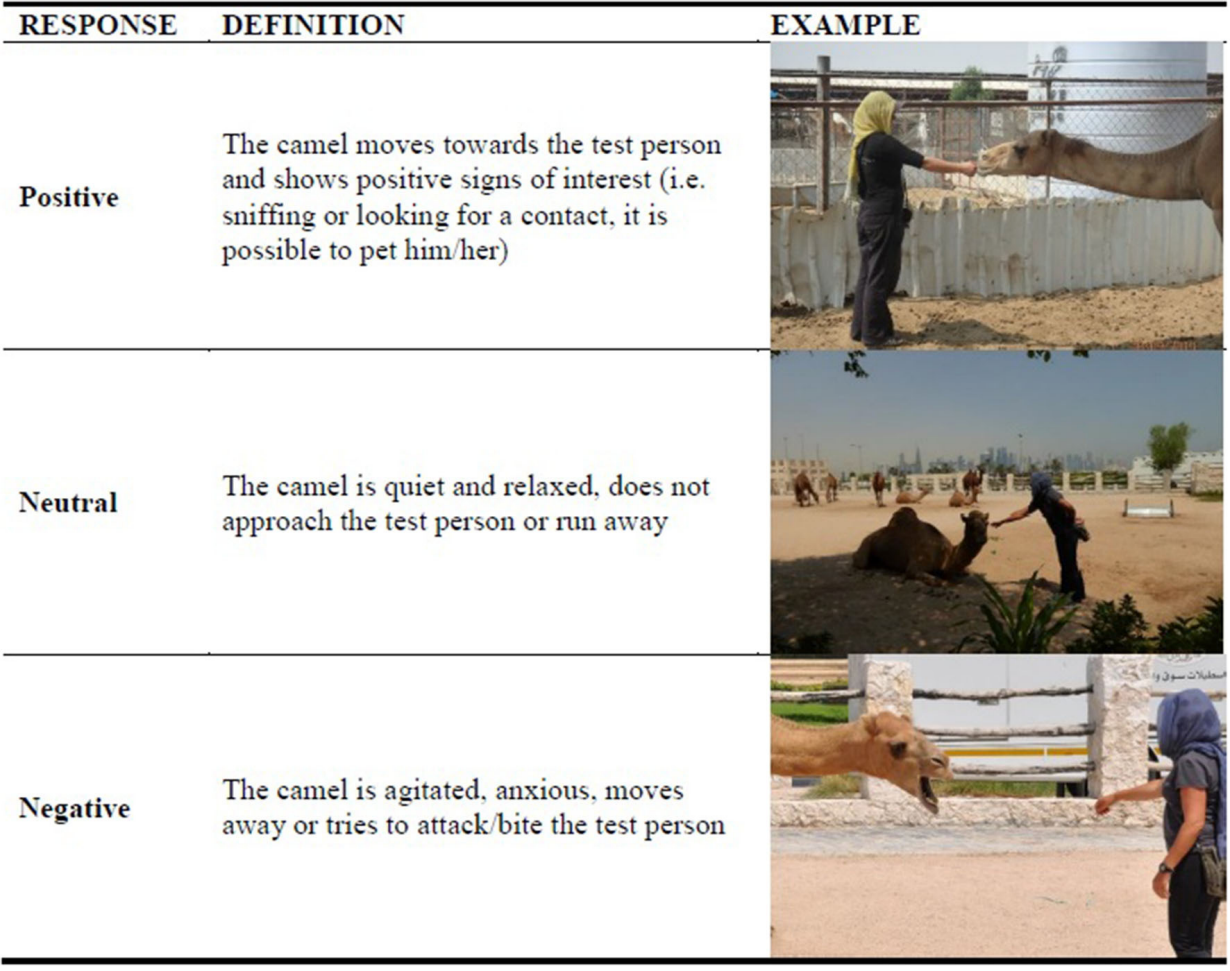

*Definitions of possible camel's responses during an approaching test*.

After the approaching test, the assessor should carry out a careful visual inspection of the camel to determine its Body Condition Score (BCS) and the presence of any disease and injuries listed in the Good health section. For the BCS, the scoring (0–5) is based on visual examination of the ribs, the ischial and coxal tuberosities, the hollow of the flank, and the recto-genital zone as suggested by Faye et al. (47) (Table 7). If the camel is hobbled or tied up, the type of hobbles, the length of the rope (and whether injuries and scars caused by them were present) should be noted down (Supplementary Figure 4). Finally, a bucket-test should be conducted as follows: a bucket is filled with 5 L of fresh and clean water and placed about 1 m far from the camel. The time the camel takes to approach the bucket after it is placed (“latency time,” in seconds) is taken using a stop-watch and the volume of water drunk (in liters) is recorded. If the camel does not drink within 60 s, the bucket is removed (Supplementary Figure 5). A categorization of these continuous measures is proposed to create a score-based index, called Thirst Index, indicating the animal's thirst (Table 10).

**Table 10:** Parameters and criteria proposed for scoring the results of a bucket-test during welfare assessment in camels.

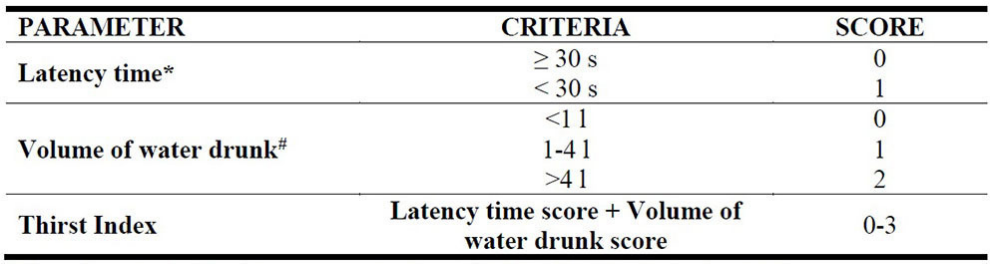

*The pre-defined thresholds categorize the latency time and the volume of water drunk into scores ranging from 0 to 2. These scores are added to obtain the Thirst Index, which can range from 0 (not thirsty) to 3 (very thirsty)*.

* *Time the camel takes to approach the bucket after it is placed, 1 min maximum*.

# *5 L maximum*.

## Discussion

This study introduced an innovative protocol for assessing welfare in camels reared in intensive or semi-intensive farming systems conceived by the idea of adapting the criteria and principles of Welfare Quality® and AWIN protocols for this species (15, 16, 18). It focused on critical aspects of farming that could negatively impact camel welfare status as indicated by the Five Freedoms paradigm, i.e., thirst, hunger, discomfort, pain, distress, and abnormal behaviors (17). However, based on the current knowledge of the camel species, the proposed tool emphasized positive welfare states and human factors according to the Five Domains Model (49, 50) and proposed indicators *ad hoc* for camels. Among the proposed welfare indicators, some were already validated in camels [e.g., BCS; (47)], others were selected based on their good feasibility, repeatability, and reliability demonstrated in other species (42, 51) and the current knowledge of camel ethology, physiology, and pathology (24–41). The proposed protocol assesses camel welfare applying a multidisciplinary approach (14, 43), suggesting several indicators for each welfare principle assessed at three different levels, namely Caretaker, Herd, and Animal (Figure 1 and Table 1). However, only further applications of the proposed welfare assessment tool on many camel farms will lead to the validation of the proposed indicators and the identification of thresholds for their acceptability as well as to the possible creation of overall welfare indices and welfare standards for camels.

“Appropriate nutrition” and “Absence of prolonged thirst” are the criteria used for the principle of Good feeding (18, 19). Hunger and thirst can occur not only when feed and water are not available, but also when they are not accessible or their quality and quantity do not meet the animals' physiological and behavioral needs (43). Thus, our protocol at the Herd level included structural and technical elements relating to feeding and watering points as well as indicators of effective availability and cleanliness of feed and water in line with the AWIN protocol (18). However, given the high environmental temperatures in which camels are usually reared, the position of the troughs in the shade/sun and the water temperature were added as measures of quality. Herd level was also implemented with some animal-based indicators of positive welfare states, such as feeding and drinking behaviors. Notwithstanding the elevated number of indicators introduced at Herd level, the assessment of welfare at this level has some limitations; firstly, it is only a snapshot of the reality, secondly, camel management and facilities may vary a lot among countries where camels are reared probably more than other livestock. The assessment at Animal level therefore becomes crucial for the evaluation of longer-term welfare conditions of camels. BCS is a robust animal-based measure for evaluating medium to long-term good feeding practices in many species (23, 52) and in camels has been validated by Faye et al. (47) and consequently applied in our protocol. Further studies could identify the welfare implications for each scoring category in camels of different age, physiological states or rearing purposes (i.e., growing camels, lactating she-camels, racing camels). As an indicator of “Absence of prolonged thirst” at Animal level, the protocol proposed a bucket-test, initially designed to evaluate thirst in horses (18). It only requires a graduated plastic container and fresh water as equipment, but biosecurity rules and good hygiene practices have to be respected to avoid the transfer of pathogens while testing animals. During a bucket-test, the latency time and the volume of water drunk can be easily recorded and scored. However, possible confounding factors could arise during the test skewing the results. In particular, different motivation factors could intervene especially if there are other animals in the pen. Furthermore, the latency time could be influenced by the temperament of the camel which could approach out of curiosity the bucket or be reluctant due to shyness or fear. For this reason, a Thrist index was proposed where differential scores were attributed to latency time and volume of water. The motivation could also be influenced by the farm system and the type of food: in intensive farm, usually forages are containing more humidity while in extensive areas, where the food is dryer, camels can be more trained to avoid drinking for several days. The results of the bucket-test, thus, should be interpreted with due caution (51) and the results of the test conducted on intensive and extensive farms should be not compared. Consequently, further studies are necessary to validate both the bucket-test and its scores as well as to develop new indicators to assess the “Absence of prolonged thirst” Criterion in camels.

“Appropriate nutrition” means that physiological and behavioral needs have to be met to ensure a good welfare state. Camels are well-known for their abilities to adapt to resource-poor environments but this could bias their welfare assessment, especially in intensive contexts. The camel, in fact, is well-adapted to the utilization of feed with low nutritional value in its natural habitat where the diet is varied and it can choose the plants by selecting the richest in water and mineral content (53). Under natural conditions, moreover, camels spend most of their time grazing and ruminating (24, 25). In intensive farms, unfortunately, the restricted feed access, as well as a diet usually less varied and poor in low-digestible feeds drastically limit these behaviors. These conditions could also have implications for rumination times, gut microbiota, and, finally, camel health (54). It is interesting to note that, according to Baraka et al. (40), 23% of farmed camels suffer from ruminal acidosis associated with low ruminal pH. Camels, unlike other herbivores, are also predisposed to diabetes mellitus and high-caloric diets can compromise their welfare (32). It is for all of these reasons that several indicators related to the feeding type, feeding strategies, and feeding behavior assume great importance in the welfare evaluation of camels reared in intensive farming systems and they have been included in our protocol. Camels have to digest many mineral salts as they are involved in the homeostatic mechanisms of thermoregulation and water retention. Including mineral supplementation in the diet has shown important effects on their metabolic profile and health (28) as well as on their milk production (33). Thus, the use of salt rocks or other supplements are important, not only for animal welfare, but also for farm productivity. Whilst the proposed protocol only registers the presence-absence of a salt block, quantitative measures of supplements, such as the number of salt blocks, the ease of access, and the physical form (i.e., solid or dissolved in drinking) could be added. Finally, not only animal-based (i.e., BCS), but also resource- and management-based measures indicating “Appropriate nutrition” should be always related to the category of animals present in the pen as nutrient requirements vary according to age, sex, and physiological status (pregnancy and lactation) (55).

“Comfort around resting,” “Thermal comfort,” and “Ease of movement” are the criteria used for the principle of Good housing (18, 19). In our protocol, the “Comfort around resting” involved measures collected at Herd level describing the space allowance, the type and quality of rubbish and bedding. Since the latter aspects may depend on managerial decisions, a question about the experience in working with animals was included at the Caretaker level. Even though these aspects may often be neglected by the caretakers on farms, they are very important to respect the natural behavior of camels and to ensure they have a clean and quiet resting site. In extensive contexts, indeed, camels show a strong attachment to sleeping sites and carefully choose the quietest places (35) but intensive farming may affect this natural resting behavior, particularly when the pen is overcrowded. The space allowance could preclude the animal from having an adequate space to rest comfortably leading to various welfare concerns. El Shoukary et al. (36) showed that overstocking resulted not only in reduced lying and rumination time but also in increased serum cortisol concentration, feed competition, aggressive behavior and production losses. Camel's resting time may also be related to the presence and type of rubbish and bedding. Rubbish may be present in camel pens and could limit the space not only for resting but also for walking. Moreover, depending on its size, rubbish could increase the risk of injury and foreign body ingestion. This is the reason why both the presence and the dimension of rubbish are listed as indicators in our protocol. The presence/absence of rubbish inside a pen depends on the caretakers; consequently, their level of general experience in working with animals was included as an indicator and could be a point on which they need to be educated. The cleanliness of bedding is also listed, although dirty assessment at Herd level could be a problem only in case of humidity as the camel feces can dry rapidly. Thus, cleanliness could be also evaluated at Animal level developing a scoring system similar to that proposed for cows (56). Finally, resting behavior is an indicator at both Herd and Animal level, because the behavioral observation becomes crucial to assess whether the camels have, like and use an adequate resting place.

The Criterion of “Thermal comfort” states that “animals should neither be too hot nor too cold” (19). Although the ability of camels to adapt to an arid climate is well-known, the prevention of prolonged heat stress is also a welfare concern for camels. Indeed, physiological adaptive mechanisms may not be adequate to alleviate heat and camels can experience heat stress (29). The primary causes of heat stress are high environmental temperatures and humidity as well as the inadequate facilities to protect the camels from these environmental challenges (29). Thus, the indicators for this Criterion not only concern the environmental parameters, but also the availability and the use of shade as well as the caretaker's experience in working with camels. Although it should be verified, we hypothesized that the knowledge acquired by the caretaker on the thermal needs of camels and the management of adverse climate conditions could optimize the allocation of resources. Heat stress in camelids can cause decreased appetite, reluctance to rise, and lethargy and even result in death of the animals. There are not many statistics on the incidence of heat stress and there is little information on its risk factors (29), but certainly, the effects of heat stress are exacerbated if it is concomitant with water deprivation (41). Some animals could also be predisposed to heat stress by other factors such as parasitism, lameness, weaning, inadequate nutrition, or obesity (29). For this reason, the indicators suggested for the principles of Good health and Good feeding can further contribute to the thermal comfort assessment. A better understanding of the camel's ethology could also be useful to identify indicators of positive experiences related to their “Thermal comfort,” as suggested in the Five Domains Model (49, 50). This Model encourages the inclusion of measures that indicate positive experiences for the animal, recognizing that acceptable animal welfare cannot be achieved only by avoiding negative states but agreeable experiences are needed as well. Therefore, minimizing the risk of thermal discomfort would not be enough. It is necessary, at the same time, to offer animals “a life worth living” providing them with opportunities to have positive experiences (50). For example, the number of animals resting or ruminating in the shade might be suggested as a positive welfare indicators although there is still no scientific evidence for this. Preference tests should be conducted to understand whether camels like resting and ruminating in the shade or under the sun.

The “Ease of movement” Criterion responds to the animal's need for an adequate space that guarantees them freedom of movement. In our protocol, a quantitative and qualitative description of the fences was proposed at Herd level as they can be a critical concern of many camel farms. The possibility of exercising was investigated at the Caretaker level, while the numbers of camels hobbled or tethered should be reported at both Herd and Animal level. Health consequences of the tools adopted for restraint are addressed below but, here, their role in the inhibition of movements is emphasized. Camels are usually calm and docile animals that, in feral conditions, live in herds moving over wide areas of land (35). However, in intensive management, it is not uncommon to find them confined in small places or even tied with short ropes and hobbled (37). This condition is a critical welfare concern, both from the point of view of freedom of movement and expressing natural behavior. Indeed, as in other species (57, 58), limited space and social isolation are the cause of chronic stress in camels which can develop stereotypies (38) and show high serum cortisol concentrations (59). Finally, movement control affected metabolism, whereby the increase in locomotory activities favored feed digestion and nutrient absorption (59). Therefore, ensuring the “Ease of movement” Criterion will also enhance camel performance.

“Absence of injuries,” “Absence of disease,” and “Absence of pain and pain induced by management procedures” are the criteria of the principle of Good health (18, 19). The remarkable resistance and adaptability of the camel can represent serious biases in the evaluation of its health. Several reports testify that camels are susceptible to a lot of diseases and can manifest more severe clinical signs than other animals (30, 31, 60). Some of these diseases mainly occur in certain periods of the year, e.g., breeding season, and could not be noticed on the day of assessment. Thus, caretakers were asked for the pathologies found in their camels during the last year in order to obtain “longitudinal” information on the incidence of the major diseases. The other critical issue is related to their remarkable ability to bear pain. They could continue to work without showing any signs of suffering and therefore medical intervention may be too late (55). In this context, early diagnosis, ability of the handlers to carry out correct evaluations and the frequency of checks assume considerable importance in guaranteeing the principle of Good health. *Ad hoc* indicators were included in our protocol but further considerations are needed. Pastoralists use several strategies to prevent and treat health conditions (61). However, the ineffectiveness of some traditional treatments, the lack of professional surgery as well as the inappropriate use of veterinary drugs and vaccines, not only compromises animal welfare, but contributes to the spread of disease and the development of drug resistance (62). Further epidemiological studies, more training of operators and a constant presence of veterinarians inside the farm would be desirable. In this regard, our protocol proposed a list of indicators at the Caretaker level to investigate the health management of camels and, in particular, to verify if veterinarians are routinely involved. However, further indicators could be added, such as the mortality and morbidity rate, indices to assess udder health, or more questions about the management of hygiene practices considering the growing importance of the camel as a dairy animal.

The measures of “Absence of pain and pain induced by management procedures” selected for the camel protocol are peculiar. Multiple indicators were selected for this Criterion taking into account the practices routinely used in camels for restraint, such as hobbles and nose-ring applications, or curative purposes, such as amputations and cauterization (61–63). Although the procedures for restraining can vary from country to country, halters, nose-rings, and hobbles are commonly used. In general, the nose piercing is a painful procedure which may also cause bacterial infections or lead to mutilation (64, 65). Hobbles, when tied too tight can not only cut the skin, leading to lesions, infection, and swelling but also cause inflammation of the tendons and lameness, and increase the risk of falls. Finally, they can reduce the circulation to the limb causing severe discomfort and pain (65). Cauterization is often practiced by caretakers to treat a wide range of diseases, including traumatic conditions, mastitis, and inflammations (61). Our measures were simplified compared to AWIN method for horses that also includes the Horse Grimace Scale (18) as not validated in camels. Thus, the development of tools for pain assessment in camels is certainly desirable and requires further studies.

“Expression of social behavior,” “Expression of other behavior,” “Good human-animal relationship,” and “Positive emotional state” are the criteria of the principle of Appropriate behavior in AWIN and Welfare Quality (18, 19). The measures of “Expression of social behavior” and “other behavior” include indicators of both negative (i.e., aggressive and other abnormal behaviors) and positive welfare states (i.e., social behaviors), and could be collected both at Herd and Animal level. The present approach could be further implemented including other behavioral tests, such as a Fear test or Avoidance distance, and a Qualitative Behavior Assessment. However, knowledge of camel behavior is still too scarce, and the concept of welfare still seems to be in its infancy, to develop more complex protocols for this species. Social behaviors must surely be considered among indicators as camels were herd animals even before domestication (35). As shown by Padalino et al. (38), social isolation and inappropriate housing increase abnormal behaviors, namely locomotor (head-shaking and pacing in a circle) and oral (self-biting and bar-mouthing) stereotypies in camels. Thus, the presence of stereotypies were selected as indicators in the present protocol. Other behaviors “away from the norm” were defined as “Abnormal Behaviors” (66, 67) and generic examples were reported as there is no literature regarding this so far. The “Good human-animal relationship” Criterion mainly involved the Animal and Caretaker level. A modified version of the approaching test was developed but it is worth noting that the camel's responses could be influenced by the farm's system. Dairy camels, for example, are usually more accustomed to the presence and manipulation by humans than camels used for fattening. Some information on the caretaker's experience in handling camels and knowledge of stress and welfare were also considered important to investigate. According to Mellor (50), several characteristics of the caretaker could affect his relationship with the animals. As shown in other species (68, 69), caretaker's knowledge, training and familiarity with the animals seem to improve empathy, attitudes, and, ultimately, their handling and welfare as well as farm productivity. As regards the Criterion of “Positive emotional state” of camels, the indicators could arise from the evaluation of their behavioral repertoire. It could be possible to suppose that appropriate time spent grazing and rumination could indicate a good welfare state. Free-living camels, indeed, moved frequently from one feeding station to another (24, 25) and their feeding behaviors were characterized by a long eating time (26, 34). However, there is no specific research and further studies still need to be done to consider these behaviors as reliable indicators of positive emotional states. The principle of Appropriate behavior has been linked to several aspects of the camel reproductive sphere (39, 70) and several physiological and pathological consequences in other species (71–73). Consequently, the assessment of indicators included in these criteria could offer possibilities to improve other aspects such as the health and reproductive management of the camel. It is worth highlighting that the assessment of welfare is multidisciplinary and health, production, and welfare are interlinked.

Overall, this study proposes a tool for assessing camel welfare on intensive or semi-intensive systems based on the literature and it is only the first step of a long process. The presented protocol has to be validated by applying it in the field and the proposed measures should also be selected, refined and aggregated to develop overall welfare indices. This protocol, therefore, needs to be implemented by camel scientists, stakeholders, and other members of the various camel industry before suggesting welfare standards for camels.

## Data Availability Statement

The original contributions generated in the study are included in the article/Supplementary Material, further inquiries can be directed to the corresponding author.

## Author Contributions

BP worked at the conception, designed the study, and edited the manuscript. BP and LM wrote the first draft of the manuscript. Both authors read and approved the submitted version.

## Conflict of Interest

The authors declare that the research was conducted in the absence of any commercial or financial relationships that could be construed as a potential conflict of interest.

## References

[1] FAO Live Animals. (2020). Available online at: http://www.fao.org/faostat/en/#data/QA/visualize (accessed June 11, 2020).

[2] Faye B Bonnet P Camel sciences and economy in the world: current situation and perspectives. 3rd ISOCARD Conf Present 29th January−1st February, 2012. Mascate: Sultanate Oman (2012). p. 2–15.

[3] Faye B The camel today: assets and potentials. Anthropozoologica. (2014) 49:167–76. 10.5252/az2014n2a01

[4] Zarrin M Riveros JL Ahmadpour A de Almeida AM Konuspayeva G Vargas-Bello-Pérez E Camelids: new players in the international animal production context. Trop Anim Health Prod. (2020) 52:903–13. 10.1007/s11250-019-02197-2 31898022

[5] Konuspayeva G Faye B Loiseau G Levieux D Lactoferrin and immunoglobulin contents in camel's milk (*Camelus bactrianus, Campus dromedarius*, and Hybrids) from Kazakhstan. J Dairy Sci. (2007) 90:38–46. 10.3168/jds.S0022-0302(07)72606-1 17183073

[6] Konuspayeva GS Camel Milk Composition and Nutritional Value. In: Omar A. Alhaj, Bernard Faye Hershey, editors. Pennsylvania: IGI global (2020). 10.4018/978-1-7998-1604-1.ch002

[7] Al haj OA Al Kanhal HA Compositional, technological and nutritional aspects of dromedary camel milk. Int Dairy J. (2010) 20:811–21. 10.1016/j.idairyj.2010.04.003

[8] Ayyash M Olaimat A Al-Nabulsi A Liu SQ. Bioactive properties of novel probiotic lactococcus lactis fermented camel sausages: cytotoxicity, angiotensin converting enzyme inhibition, antioxidant capacity, and antidiabetic activity. Food Sci Anim Resour. (2020) 40:155–71. 10.5851/kosfa.2020.e1 32161912 PMC7057035

[9] Khatoon H Ikram R Anser H Naeem S Khan SS Fatima S. Investigation of anti-inflammatory and analgesic activities of camel milk in animal models. Pak J Pharm Sci. (2019) 32:1879–83. 31680087

[10] Gebremichael B Girmay S Gebru M Camel milk production and marketing: pastoral areas of Afar, Ethiopia. Pastoralism. (2019) 9 10.1186/s13570-019-0147-7

[11] Nagy P Juhasz J. Review of present knowledge on machine milking and intensive milk production in dromedary camels and future challenges. Trop Anim Health Prod. (2016) 48:915–26. 10.1007/s11250-016-1036-3 26992732

[12] Pastrana CI González FJN Ciani E Capote CJB Bermejo JVD. Effect of research impact on emerging camel husbandry, welfare and social-related awareness. Animals. (2020) 10:780. 10.3390/ani10050780 32365928 PMC7277471

[13] OIE Terrestrial Animal Health Code. World Organ Anim Heal (2019). Available online at: https://www.oie.int/en/standard-setting/terrestrial-code/access-online/ (accessed June 15, 2020).

[14] Blokhuis HJ Veissier I Miele M Jones B The welfare quality® project and beyond: safeguarding farm animal well-being. Acta Agric Scand A Anim Sci. (2010) 60:129–40. 10.1080/09064702.2010.523480

[15] Welfare Quality Network Welfare Quality® Project. (2009). Available online at: http://www.welfarequality.net/en-us/news/assessment-protocols/ (accessed June 15, 2020).

[16] European Animal Welfare Indicators Project (AWIN) Animal Welfare Science Hub. (2020). Available online at: http://www.animalwelfarehub.com/about-us (accessed June 15, 2020).

[17] Farm Animal Welfare Council Five Freedoms. Farm Anim Welf Counc (2009) 5 Available online at: http://www.fawc.org.uk/freedoms.htm (accessed June 15, 2020).

[18] AWIN AWIN Welfare Assessment Protocol for Horses. AWIN (2015). p. 1–80. 10.13130/AWIN_HORSES_2015

[19] Welfare Quality® Welfare Quality® Assessment Protocol for Cattle. Lelystad: Welfare Quality® Consortium (2009).

[20] Main DCJ Kent JP Wemelsfelder F Ofner E Tuyttens FAM. Applications for methods of on-farm welfare assessment. Anim Welf . (2003) 12:523–8. Available online at: http://www.ingentaconnect.com/content/ufaw/aw/2003/00000012/00000004/art0001119319712

[21] Battini M Barbieri S Vieira A Can E Stilwell G Mattiello S. The use of qualitative behaviour assessment for the on-farm welfare assessment of dairy goats. Animals. (2018) 8:123. 10.3390/ani8070123 30029507 PMC6071242

[22] Dalla Costa E Dai F Lebelt D Scholz P Barbieri S Canali E Welfare assessment of horses: the AWIN approach. Anim Welf . (2016) 25:481–8. 10.7120/09627286.25.4.481

[23] Dunston-Clarke E Willis RS Fleming PA Barnes AL Miller DW Collins T. Developing an animal welfare assessment protocol for livestock transported by sea. Animals. (2020) 10:705. 10.3390/ani10040705 32316532 PMC7222738

[24] Khan B Leteef M Bilal M Iqbal A Hassan R A study on some of the activity patterns of Camelus dromedarius maintained in Thal area of the Punjab Pakistan. Pak J Agric Sci. (1998) 33:67–72.

[25] Dereje M Udén P The browsing dromedary camel: I. Behaviour, plant preference and quality of forage selected. Anim Feed Sci Technol. (2005) 121:297–308. 10.1016/j.anifeedsci.2005.01.017

[26] Hedi A Khemais K Intake, digestion and feeding behaviour of the one-humped camel stall-fed straw-based diets. Livest Res Rural Dev. (1990) 2:2.

[27] Elmahdi B Sallmann HP Fuhrmann H Von Engelhardt W Kaske M. Comparative aspects of glucose tolerance in camels, sheep, and ponies. Comp Biochem Physiol A Physiol. (1997) 118:147–51. 10.1016/S0300-9629(96)00449-5 9243815

[28] Faye B Ratovonanahary M Chacornac JP Soubre P. Metabolic profiles and risks of diseases in camels in temperate conditions. Comp Biochem Physiol Part A Physiol. (1995) 112:67–73. 10.1016/0300-9629(95)00088-O 7553338

[29] Norton PL Gold JR Russell KE Schulz KL Porter BF. Camelid heat stress: 15 cases (2003-2011). Can Vet J. (2014) 55:992–6. 25320390 PMC4187376

[30] Agab H Abbas B Epidemiological studies on camel diseases in eastern Sudan. World Anim Rev. (1999) 92:42–51.

[31] Sazmand A Joachim A. Parasitic diseases of camels in Iran (1931-2017)—A literature review. Parasite. (2017) 24:21. 10.1051/parasite/2017024 28617666 PMC5479402

[32] Al Haj Ali M Nyberg F Chandranath SI Ponery AS Adem A Adeghate E. Effect of high-calorie diet on the prevalence of diabetes mellitus in the one-humped camel (*Camelus dromedarius*). Ann N Y Acad Sci. (2006) 1084:402–10. 10.1196/annals.1372.034 17151318

[33] Onjoro PA Njoka-Njiru EN Ottaro JM Simon A Schwartz HJ Effects of mineral supplementation on milk yield of free-ranging camels (*Camelus dromedarius*) in northern Kenya. Asian-Australasian J Anim Sci. (2006) 19:1597–602. 10.5713/ajas.2006.1597

[34] Aubè L Fatnassi M Monaco D Khorchani T Lacalandra GM Hammadi M. Daily rhythms of behavioral and hormonal patterns in male dromedary camels housed in boxes. PeerJ. (2017) 5:e3074. 10.7717/peerj.3074 28367365 PMC5374969

[35] Schulte N Klingel H Herd structure, leadership, dominance and site attachment of the camel, *Camelus dromedarius*. Behaviour. (1991) 118:103–14. 10.1163/156853991X00229

[36] El Shoukary RD Osman A Mohammed A Effects of stocking density on some behavioral and some blood biochemical parameters in camel during the rut period. Egypt J Vet Sci. (2020) 51:253–62. 10.21608/ejvs.2020.24526.1153

[37] Padalino B Monaco D Lacalandra M Male camel behavior and breeding management strategies: how to handle a camel bull during the breeding season? Emir J Food Agric. (2015) 27:338–49. 10.9755/ejfa.v27i4.19909

[38] Padalino B Aubé L Fatnassi M Monaco D Khorchani T Hammadi M. Could dromedary camels develop stereotypy? The first description of stereotypical behaviour in housed male dromedary camels and how it is affected by different management systems. PLoS ONE. (2014) 9:e89093. 10.1371/journal.pone.0089093 24586522 PMC3929658

[39] Fatnassi M Padalino B Monaco D Aubé L Khorchani T Lacalandra GM. Effect of different management systems on rutting behavior and behavioral repertoire of housed Maghrebi male camels (*Camelus dromedarius*). Trop Anim Health Prod. (2014) 46:861–7. 10.1007/s11250-014-0577-6 24659302

[40] Baraka TA El-Sherif MT Kubesy AA Illek J Clinical studies of selected ruminal and blood constituents in dromedary camels affected by various diseases. Acta Vet Brno. (2000) 69:61–8. 10.2754/avb200069010061

[41] Bouâouda H Achâaban MR Ouassat M Oukassou M Piro M Challet E. Daily regulation of body temperature rhythm in the camel (*Camelus dromedarius*) exposed to experimental desert conditions. Physiol Rep. (2014) 2:e12151. 10.14814/phy2.12151 25263204 PMC4270234

[42] Scott EM Nolan AM Fitzpatrick JL Conceptual and methodological issues related to welfare assessment: a framework for measurement. Acta Agric Scand A Anim Sci. (2001) 51:5–10. 10.1080/090647001316922983

[43] Battini M Vieira A Barbieri S Ajuda I Stilwell G Mattiello S. Invited review: animal-based indicators for on-farm welfare assessment for dairy goats. J Dairy Sci. (2014) 97:6625–48. 10.3168/jds.2013-7493 25242423

[44] Menchetti L Monaco D Ziani A Padalino B Camel welfare: the first survey on camel caretakers' perspective. J Camelid Sci. (2020) Accepted paper.

[45] Altmann J. Observational study of behavior: sampling methods. Behaviour. (1974) 49:227–67. 10.1163/156853974X00534 4597405

[46] AWIN Goats AWIN Welfare Assessment Protocol. AWIN (2015). 10.13130/AWIN_GOATS_2015

[47] Faye B Bengoumi M Cleradin A Tabarani A Chilliard Y Body condition score in dromedary camel: a tool for management of reproduction. Emirates J Food Agric. (2001) 13:1–6. 10.9755/ejfa.v12i1.5193

[48] Wulf M Aurich J May AC Aurich C Sex differences in the response of yearling horses tohandling by unfamiliar humans. J Vet Behav Clin Appl Res. (2013) 8:238–44. 10.1016/j.jveb.2012.09.002

[49] Mellor DJ Beausoleil NJ. Extending the “Five Domains” model for animal welfare assessment to incorporate positive welfare states. Anim Welf . (2015) 24:241–53. 10.7120/09627286.24.3.24127669313

[50] Mellor DJ. Updating animal welfare thinking: moving beyond the “five freedoms” towards “A life worth living.” *Animals*. (2016) 6:21. 10.3390/ani6030021 27102171 PMC4810049

[51] Dalla Costa E Murray L Dai F Canali E Minero M Equine on-farm welfare assessment: a review of animal-based indicators. Anim Welf . (2014) 23:323–41. 10.7120/09627286.23.3.323

[52] Menchetti L Brecchia G Cardinali R Polisca A Boiti C Feed restriction during pregnancy: effects on body condition and productive performance of primiparous rabbit does. World Rabbit Sci. (2015) 23:1–8. 10.4995/wrs.2015.1703

[53] Laudadio V Tufarelli V Dario M Hammadi M Seddik MM Lacalandra GM. A survey of chemical and nutritional characteristics of halophytes plants used by camels in Southern Tunisia. Trop Anim Health Prod. (2009) 41:209–15. 10.1007/s11250-008-9177-7 18500670

[54] Samsudin AA Wright AD Al Jassim R. The effect of fibre source on the numbers of some fibre-degrading bacteria of Arabian camel's (*Camelus dromedarius*) foregut origin. Trop Anim Health Prod. (2014) 46:1161–6. 10.1007/s11250-014-0621-6 24898095

[55] Previti A Guercio B Passantino A. Protection of farmed camels (*Camelus dromedarius*): welfare problems and legislative perspective. Anim Sci J. (2016) 87:183–9. 10.1111/asj.12446 26260977

[56] Faye B Barnouin J Objectivation de la propreté des vaches laitières et des stabulations. L'indice de propreté. Bull Tech Cent Rech Zootech Vétérinaires Theix. (1985) 59:61–7.

[57] Beerda B Schilder MBH Van Hooff JARAM De Vries HW Mol JA Chronic stress in dogs subjected to social and spatial restriction. I. Behavioral responses. Physiol Behav. (1999) 66:233–42. 10.1016/S0031-9384(98)00289-3 10336149

[58] Cooper J McGreevy P Stereotypic behaviour in the stabled horse: causes, effects and prevention without compromising horse welfare. In: Waran N, editor. The Welfare of Horses. Animal Welfare, vol 1. Dordrecht: Springer (2007).

[59] El-Shoukary RD Nasreldin N Osman AS Hashem NM Saadeldin IM Swelum AA. Housing management of male dromedaries during the rut season: effects of social contact between males and movement control on sexual behavior, blood metabolites and hormonal balance. Animals. (2020) 10:1–11. 10.3390/ani10091621 32927818 PMC7552277

[60] Abbas B Omer OH Review of infectious diseases of the camel. Vet Bull. (2005) 75:1N−16N.

[61] Volpato G Lamin Saleh MS Nardo A. Ethnoveterinary of Sahrawi pastoralists of Western Sahara: camel diseases and remedies. J Ethnobiol Ethnomed. (2015) 11:54. 10.1186/s13002-015-0040-4 26087846 PMC4477503

[62] Basheir BO ElMalik KH Abdelgadir AE Gameel AAR Traditional and modern practices in the diagnosis, treatment and prevention of animal diseases in South Kordofan State, Sudan. J Cell Anim Biol. (2012) 6:213–25. 10.5897/JCAB11.066

[63] Ranjan R Tuteja FC Kashinath Patil NV A survey on traditional practices adopted for restraining camel in Rajasthan. Indian J Anim Sci. (2017) 87:118–21. Available online at: http://epubs.icar.org.in/.../66940

[64] Stafford KJ Mellor DJ Painful husbandry procedures in livestock and poultry. In: Grandin T, editor. Improving Animal Welfare: A Practical Approach. London: CABI Publishing p. 337.

[65] Rayner EL Airikkala-Otter I Susheelan A Mellanby RJ Meunier NV Gibson A. Prevalence of mutilations and other skin wounds in working donkeys in Tamil Nadu, India. Vet Rec. (2018) 183:450. 10.1136/vr.104863 30121636

[66] Cooper JJ Mason GJ. The identification of abnormal behaviour and behavioural problems in stabled horses and their relationship to horse welfare: a comparative review. Equine Vet J Suppl. (1998) 27:5–9. 10.1111/j.2042-3306.1998.tb05136.x 10484995

[67] Mason GJ Stereotypies: a critical review. Anim Behav. (1991) 41:1015–37. 10.1016/S0003-3472(05)80640-2

[68] Coleman GJ Hemsworth PH. Training to improve stockperson beliefs and behaviour towards livestock enhances welfare and productivity. OIE Rev Sci Tech. (2014) 33:131–7. 10.20506/rst.33.1.2257 25000785

[69] des Roches A de B Veissier I Boivin X Gilot-Fromont E Mounier L. A prospective exploration of farm, farmer, and animal characteristics in human-animal relationships: an epidemiological survey. J Dairy Sci. (2016) 99:5573–85. 10.3168/jds.2015-10633 27085406

[70] Bhakat C Raghavendra S Sahani MS Effect of different management conditions on rutting behavior of Indian dromedary camel. Emirates J Food Agric. (2005) 17:1–13. 10.9755/ejfa.v12i1.5085

[71] Alexander SL Irvine CHG Livesey JH Donald RA. Effect of isolation stress on concentrations of arginine vasopressin, α-melanocyte-stimulating hormone and ACTH in the pituitary venous effluent of the normal horse. J Endocrinol. (1988) 116:325–34. 10.1677/joe.0.1160325 2832503

[72] Herskin MS Munksgaard L Andersen JB. Effects of social isolation and restraint on adrenocortical responses and hypoalgesia in loose-housed dairy cows. J Anim Sci. (2007) 85:240–7. 10.2527/jas.2005-346 17179562

[73] Mumtaz F Khan MI Zubair M Dehpour AR. Neurobiology and consequences of social isolation stress in animal model-A comprehensive review. Biomed Pharmacother. (2018) 105:1205–22. 10.1016/j.biopha.2018.05.086 30021357

