# Supplementary material for: The First Protocol for Assessing Welfare of Camels
Source: Front Vet Sci. 2021 Jan 28;7:631876. doi: 10.3389/fvets.2020.631876 (PMC7876076; doi:10.3389/fvets.2020.631876)
Supplement: Supplementary file 1 [file Data_Sheet_1.docx]

Supplementary Material

## Supplementary Table 1. Ethogram of the behaviors reported in the recording sheets for camel welfare assessment.

| **Behavior** | **Definitions** |
| --- | --- |
| Resting | The camel sits in sternal or lateral recumbency. |
| Standing quietly | The camel stands on its four feet and appears calm and relaxed. |
| Social behavior | The camel comes into physical contact (e.g. touches, sniffs, allo-grooming) with another camel. |
| Feeding | The camel takes food into its mouth (hay or concentrate), chews and swallows it. |
| Rumination | A bolus goes back into its mouth and the camel chews it. |
| Aggressive behavior | The camel attempts or bites/pushes or kicks another camel. |
| Stereotypical behavior | The camel shows locomotor (pacing in a circle or head-shaking) or oral (self-biting, self-mutilation, bar-mouthing) stereotypies (1) |
| Other abnormal behaviors | The camel shows other behaviors “away from the normal” (2) including signs of fear, frustration, uneasiness or anxiety (e.g. avoidance, over-reaction to minor environmental changes, motor inhibition, restless, violent escape reactions). |

1. Padalino B, Aubé L, Fatnassi M, Monaco D, Khorchani T, Hammadi M, Lacalandra GM. Could dromedary camels develop stereotypy? The first description of stereotypical behaviour in housed male dromedary camels and how it is affected by different management systems. *PLoS One* (2014) **9**: doi:10.1371/journal.pone.0089093

2. Mason Georgia J. Stereotypies: a critical review. *Anim Behav* (1991) **41**:1015–1037.

## Supplementary Figures

**Supplementary Figure 1.** The flow of steps proposed for camel welfare assessment. The on-farm visit should start with a meeting with the stable manager/caretaker and with the recording of data from outside the pen. The entrance into the pen coincides with the approaching test, is followed by the visual inspection of the camels, and finishes with the bucket test. Measurements of resources and facilities should be carried out without disturbing the animals. BCS: Body Condition Score.

| 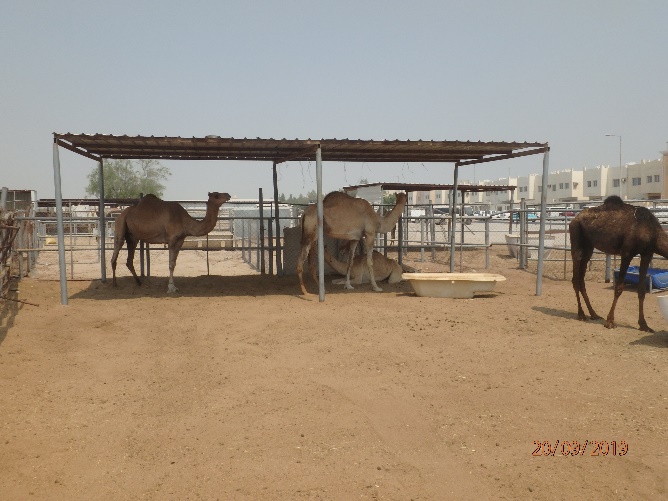  A | 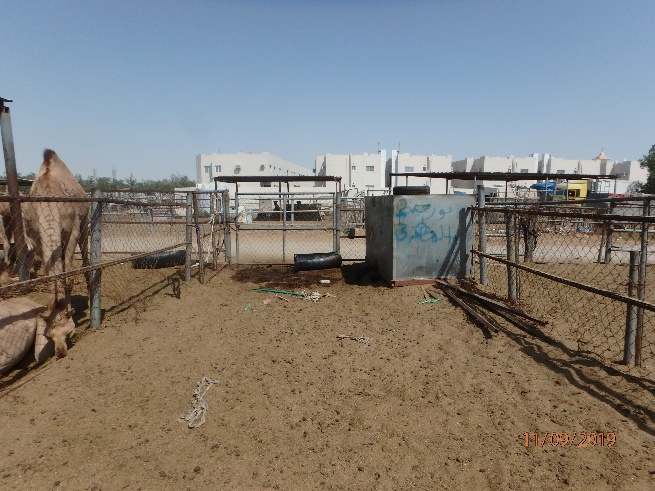  B |
| --- | --- |
| 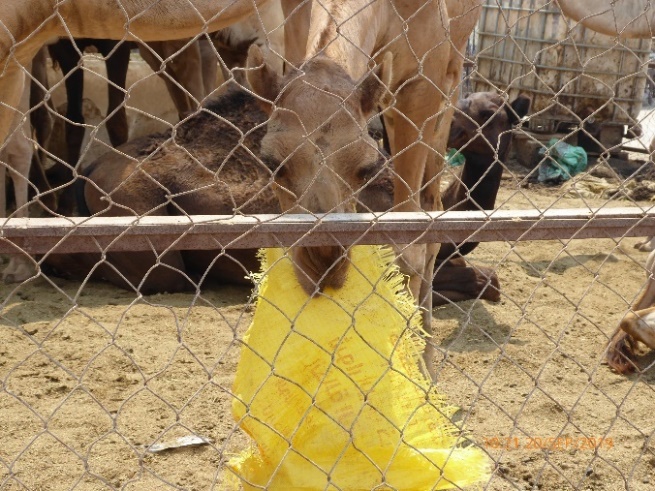  C | 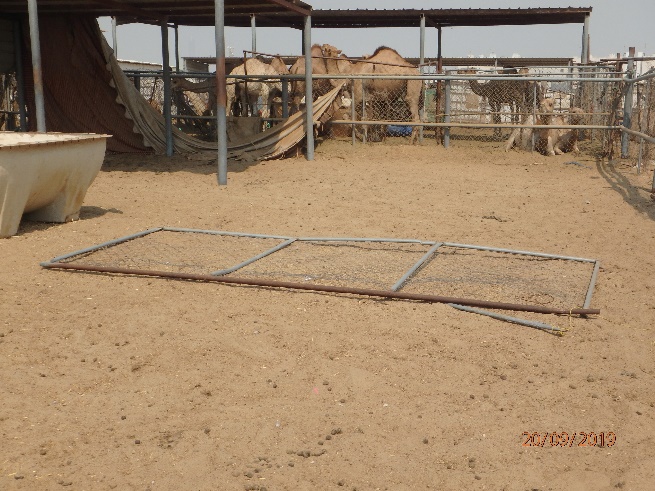  D |

**Supplementary Figure 2.** Rubbish. The rubbish should be scored as “No rubbish” **(A)**, “Small” **(B)**, “Medium” **(C)** or “Large” size **(D)** according to its dimensions. Indeed, depending on its size, rubbish could increase the risk of foreign body ingestion **(B, C)** or injury **(D)** and limit the space for resting and walking **(D)**. The red arrows indicate the different kinds of rubbish according to the proposed classification.

| 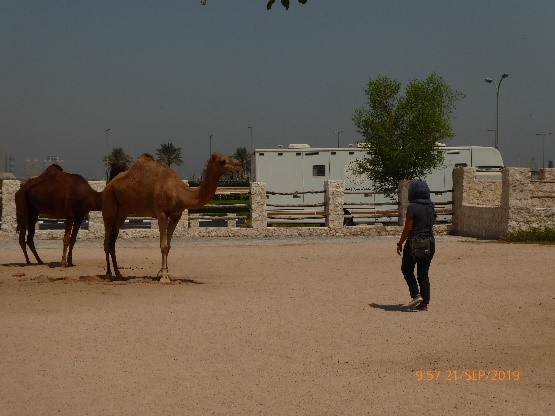  A | 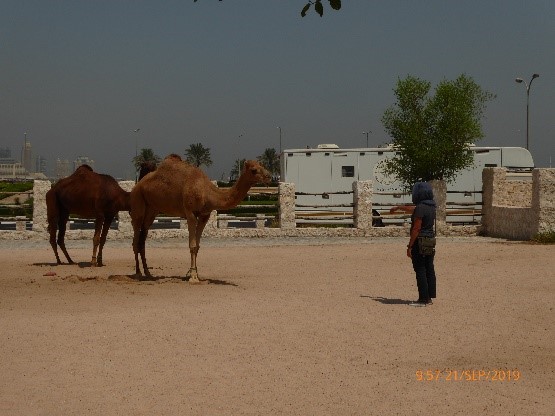  B | 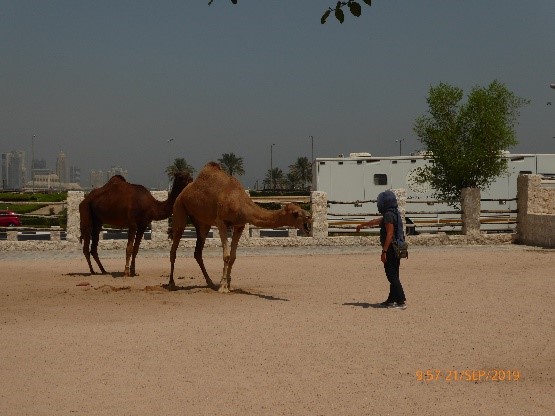  C |
| --- | --- | --- |
| 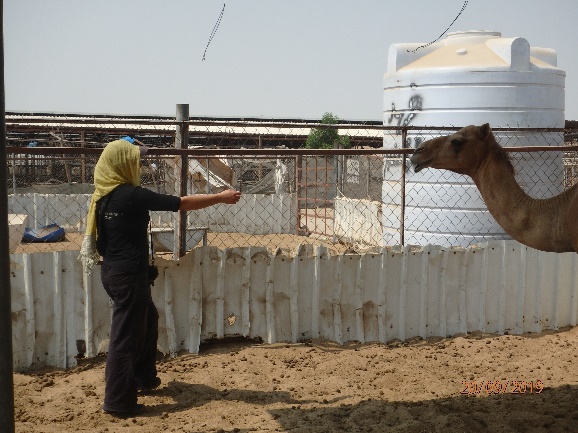  D | 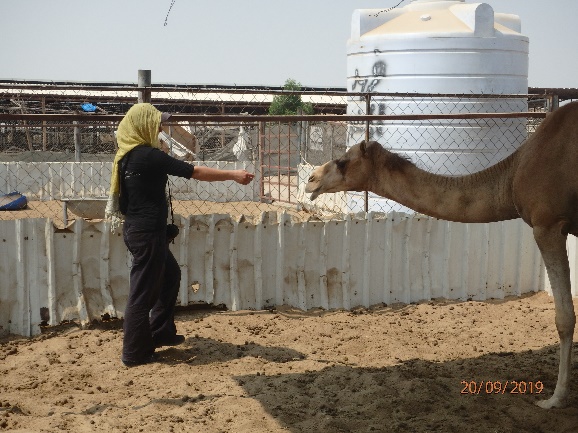  E | 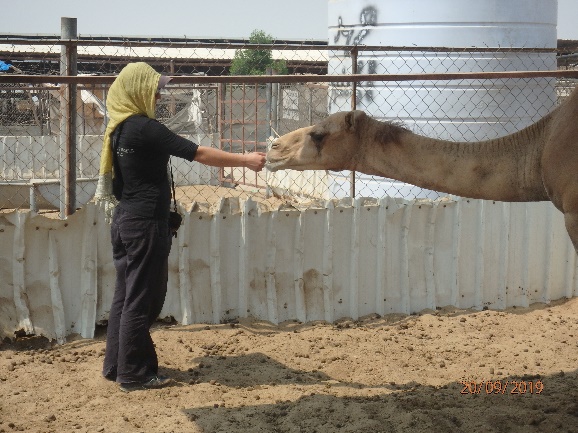  F |

**Supplementary Figure 3.** Two approaching tests. The tester (an unfamiliar person) enters into the pen where the camel is kept and approaches the camel slowly, one step at a time **(A, B, D, and E)**. The test is stopped if the camel shows avoidance or aggressive behavior **(C)** or when the tester can approach the camel and put a hand close to the nose of the camel **(F)**.

| 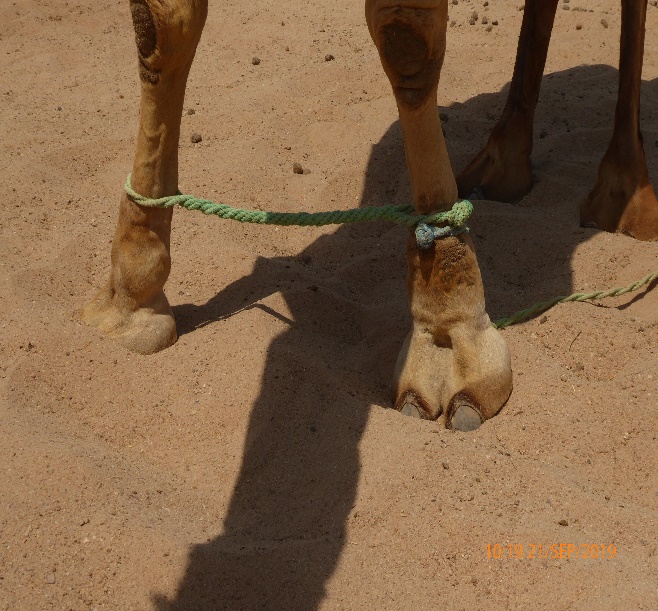  A | 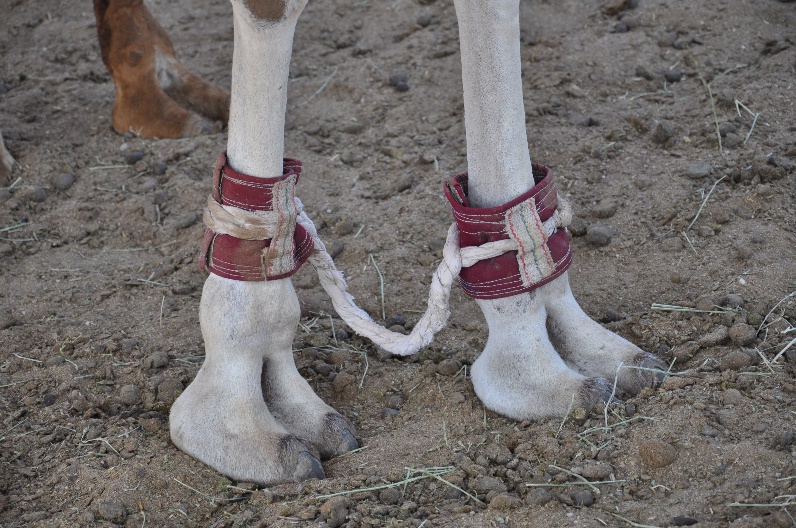  B |
| --- | --- |
| 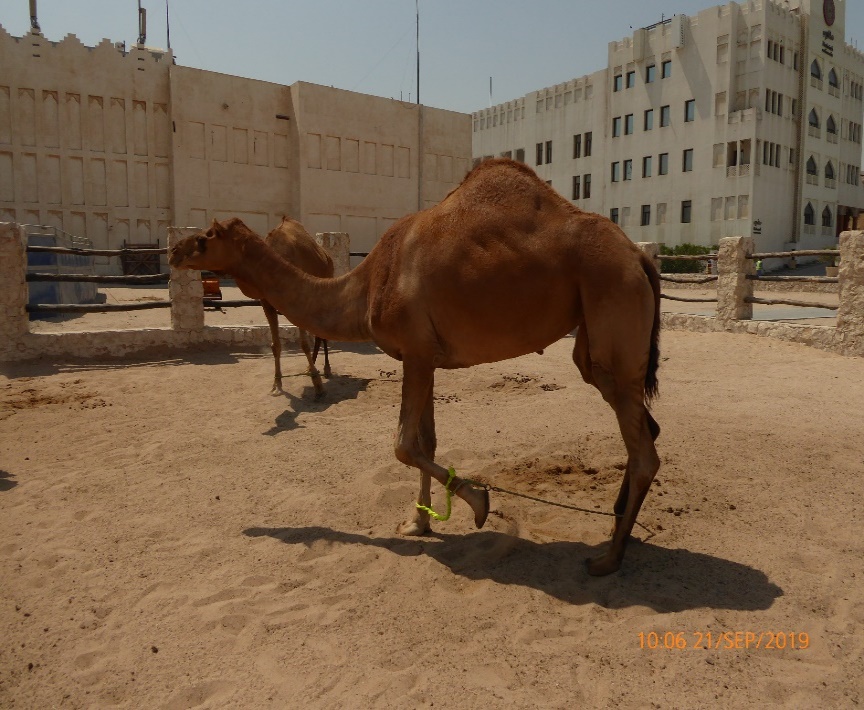  C | 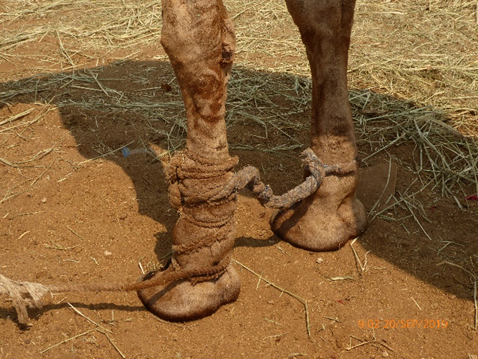  D |
| 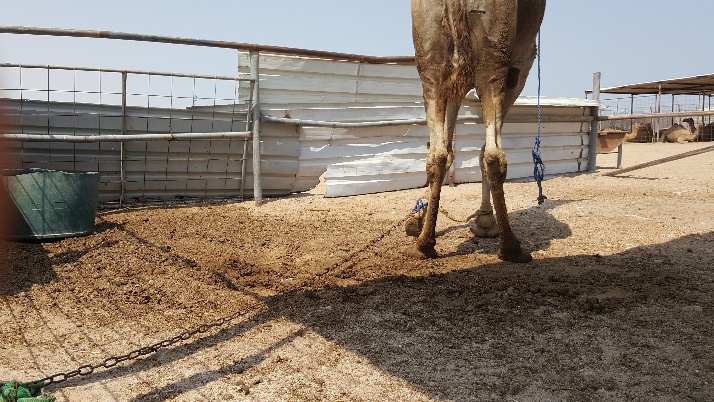  E | 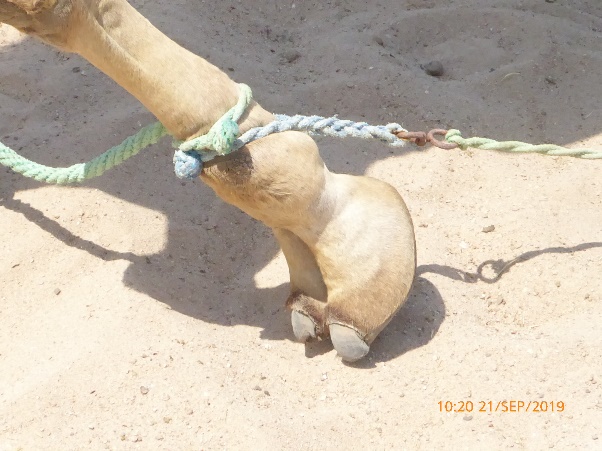  F |

**Supplementary Figure 4.** Ropes and hobbles. Hobbles cutting skin, leading to lesions and swelling **(A)**. The use of pads under the hobbles reducing the risk of injury **(B)**. Short ropes or incorrect tethering **(C-F)** that may cause falls, injuries, and bone fractures.

| 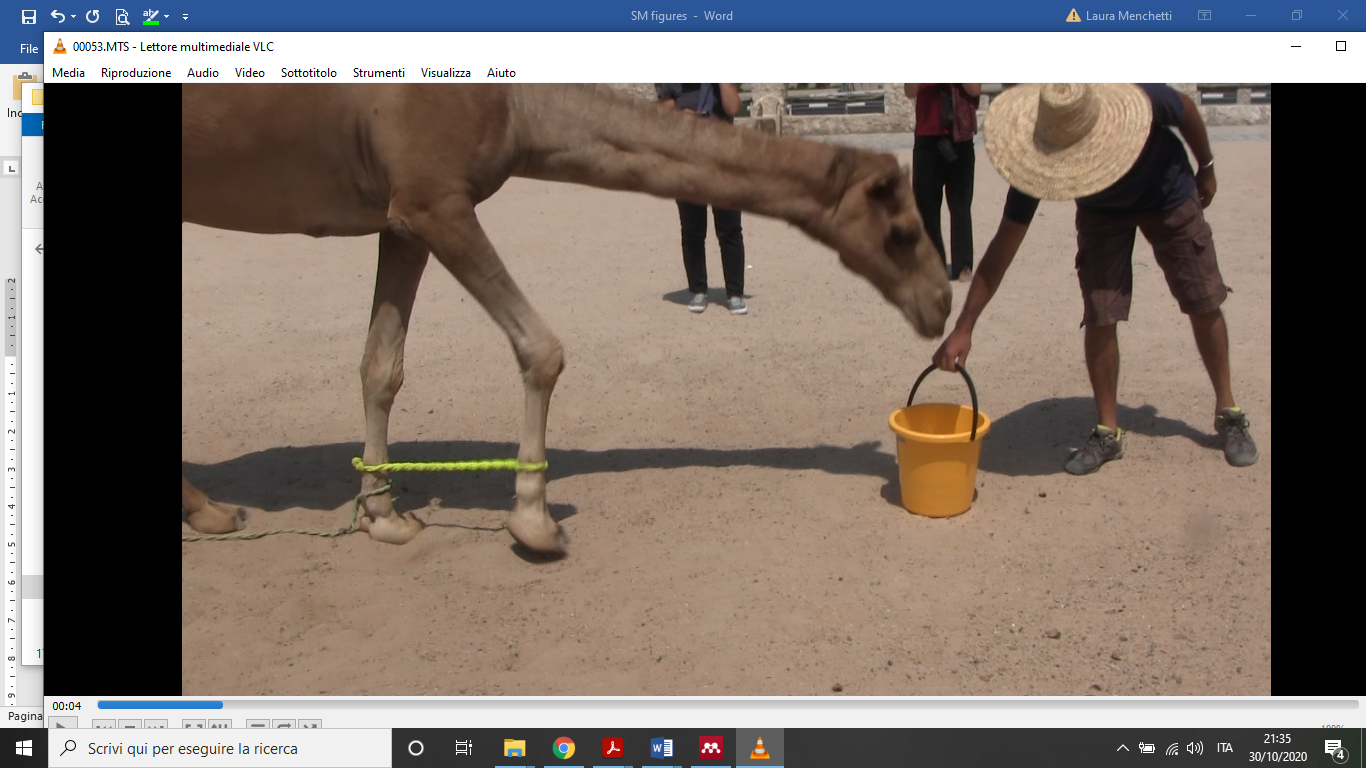  A | 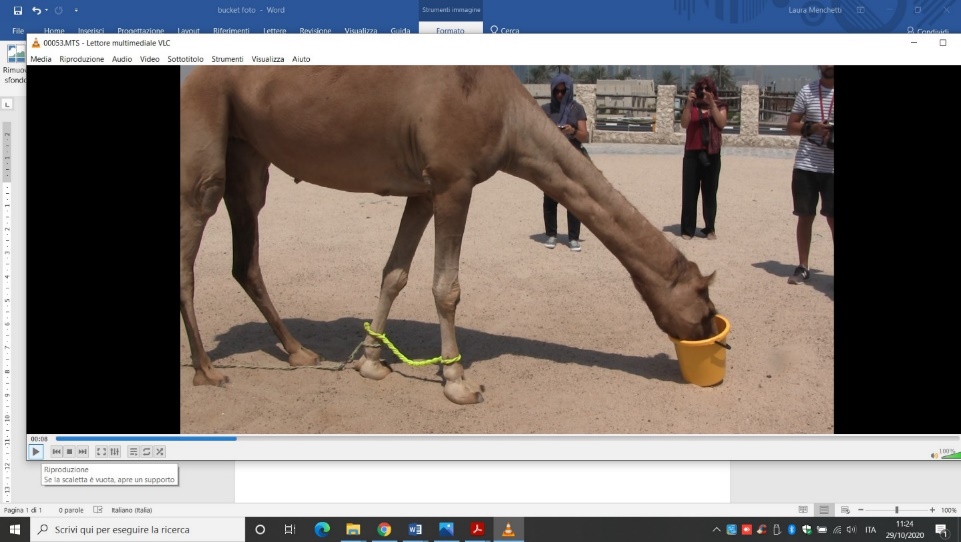  B | 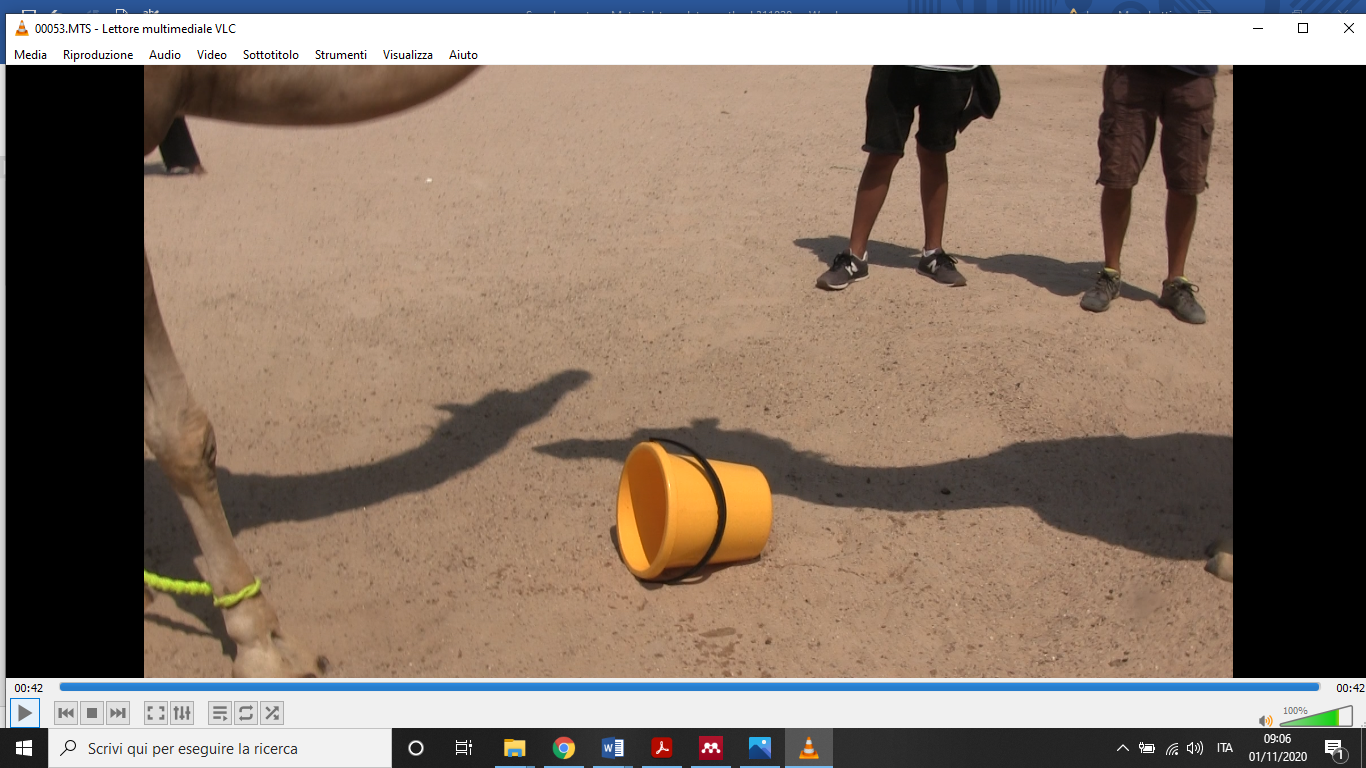  C |
| --- | --- | --- |

**Supplementary Figure 5.** Bucket test. A tester places a bucket filled with fresh and clean water about 1 meter away from the camel **(A)**. The time the camel takes to approach the bucket is recorded **(B)** and at the end of the test, the bucket is removed and the volume of water drunk is measured **(C)**.
